# Clinical Impact of Neoadjuvant Therapy for Resectable Pancreatic Ductal Adenocarcinoma: A Single-Center Retrospective Study

**DOI:** 10.1245/s10434-024-16851-z

**Published:** 2025-01-23

**Authors:** Gaku Shimane, Minoru Kitago, Hiroshi Yagi, Yuta Abe, Yasushi Hasegawa, Shutaro Hori, Masayuki Tanaka, Junya Tsuzaki, Yoichi Yokoyama, Yohei Masugi, Ryo Takemura, Yuko Kitagawa

**Affiliations:** 1 Department of Surgery, Keio University School of Medicine, Tokyo, Japan; 2 Department of Radiology, Keio University School of Medicine, Tokyo, Japan; 3 Division of Diagnostic Pathology, Keio University School of Medicine, Tokyo, Japan; 4 Biostatistics Unit, Clinical and Translational Research Center, Keio University Hospital, Tokyo, Japan

**Keywords:** Resectable pancreatic cancer, Upfront surgery, Neoadjuvant therapy, Biomarker, Intention-to-treat analysis

## Abstract

**Background:**

Neoadjuvant therapy is recommended for treating resectable pancreatic ductal adenocarcinoma (PDAC); however, its appropriate use in patients with resectable PDAC remains debatable.

**Objective:**

This study aimed to identify independent poor prognostic factors and evaluate the clinical significance of neoadjuvant therapy in patients with resectable PDAC.

**Methods:**

We retrospectively reviewed consecutive patients diagnosed with resectable PDAC at our institute between January 2003 and December 2022. We analyzed poor prognostic factors at the time of diagnosis in patients who underwent upfront surgery using the Cox proportional hazards model for overall survival (OS). The prognostic score was calculated by adding the individual prognostic factor scores.

**Results:**

Overall, 359 patients were included in this study, with 308 patients undergoing upfront surgery and the remaining 51 patients receiving neoadjuvant therapy. The R0 resection rate was significantly higher in the neoadjuvant therapy group (70.6%) than in the upfront surgery group (64.0%). Multivariate analysis in the upfront surgery group revealed the following independent poor prognostic factors: tumor size ≥ 35 mm, serum albumin level ≤ .5 g/dL, neutrophil-to-lymphocyte ratio ≥ 3.5, carbohydrate antigen 19-9 level ≥ 250 U/mL, and Duke pancreatic monoclonal antigen type 2 level ≥ 750 U/mL. Among patients with prognostic scores of 0–1 (*n* = 263), the intention-to-treat OS did not significantly differ between the neoadjuvant therapy and upfront surgery groups. Among those patients with a prognostic score of ≥ 2 (*n* = 96), the neoadjuvant therapy group had significantly longer intention-to-treat OS than the upfront surgery group.

**Conclusions:**

Prognostic score-based stratification can help identify patients who could benefit from neoadjuvant therapy.

**Supplementary Information:**

The online version contains supplementary material available at 10.1245/s10434-024-16851-z.

Pancreatic ductal adenocarcinoma (PDAC) is among the most aggressive cancers. It is the fourth leading cause of cancer-related mortality in the United States owing to the difficulty in detecting it early.^SPS:refid::bib11^ Its associated 5-year survival rate is approximately 10%.^2^ About 40% of patients experience early recurrence after resection,^3^ and most already exhibit systemic disease at the time of surgery.

Neoadjuvant therapy has gained attention as a PDAC treatment. The advantages of neoadjuvant therapy over upfront surgery followed by adjuvant chemotherapy include high tolerance, good local control, elimination of radiologically occult metastatic disease, and prevention of early recurrence;^4, 5^ it is a standard treatment for borderline resectable PDAC.^4, 6^ However, neoadjuvant therapy for resectable PDAC has not consistently demonstrated a survival advantage in prospective clinical trials and the supporting evidence is limited.^4, 5, 7-9^ Consequently, the National Comprehensive Cancer Network (NCCN) guidelines recommend upfront surgery as first-line treatment in most patients with resectable PDAC;^1^ neoadjuvant therapy is recommended only when lymph node enlargement, extreme weight loss, and severe pain are present.^1^

Prospective evidence is lacking on whether upfront systemic chemotherapy is beneficial. High early recurrence rates and translational evidence reveal that micrometastasis occurs in most patients with resectable PDAC.^10^ The International Study Group of Pancreatic Surgery and International Association of Pancreatology have proposed anatomical, biological, and conditional classifications.^11, 12^ The anatomical classification alone may not sufficiently reflect cancer aggressiveness, and considering the biological classification is important. Therefore, incorporating the biological and conditional classifications into decision making for resectable PDAC management may help physicians identify neoadjuvant therapy candidates.

This study aimed to evaluate prognostic clinical factors in patients with resectable PDAC who underwent upfront surgery and identify potential beneficiaries of neoadjuvant therapy.

## Methods

### Participants and Data Collection

We retrospectively analyzed prospectively collected data of consecutive patients radiologically diagnosed with resectable PDAC and treated at Keio University Hospital (Tokyo, Japan) between May 2003 and December 2022. Patients diagnosed with borderline resectable PDAC, unresectable PDAC, or intraductal papillary mucinous carcinoma were excluded.

Tumor resectability was assessed using dynamic contrast-enhanced multidetector computed tomography (CT). Resectability was determined according to the NCCN guidelines.

Tumors that were not in direct contact with major arteries or the portal vein/superior mesenteric vein (PV/SMV), as well as tumors with < 180° of contact with the PV/SMV, were classified as resectable.^1^ More than two experienced radiologists conducted image interpretation, and a multidisciplinary team comprising hepatobiliary-pancreatic surgeons, internal physicians, oncologists, and radiologists assessed tumor resectability.

We analyzed patient demographics; clinicopathological findings at diagnosis; radiological findings, such as tumor location, tumor size, resectability, and lymph node enlargement; tumor marker levels, specifically carbohydrate antigen 19-9 (CA19-9) and Duke pancreatic monoclonal antigen type 2 (DUPAN-2) levels; laboratory findings, including serum albumin level and neutrophil-to-lymphocyte ratio; and pathological findings, including tumor size, differentiation, lymphovascular invasion, perineural invasion, nodal involvement, and surgical resection margins.

We used the opt-out informed consent method. The Ethics Committee of Keio University School of Medicine approved this study (approval number: 20120389), which was conducted in accordance with the Declaration of Helsinki, 1975.

### Chemotherapy Protocols

Some patients with T3/4 resectable and borderline resectable PDAC received neoadjuvant therapy based on the attending physician’s decision. Previous reports from our institute describe neoadjuvant chemoradiotherapy protocols.^13^ The neoadjuvant chemoradiotherapy schedule for 2003–2012 comprised a 28-day concurrent administration of 5-fluorouracil-based chemotherapy and radiotherapy (40.0 Gy in 20 fractions).^13^ We administered 5-fluorouracil (300 mg/day) on days 6, 13, 20, and 27; cisplatin (10 mg/day) on days 5, 12, 19, and 26; and mitomycin (4 mg/day) on days 6, 13, 20, and 27, in addition to continuous peripheral venous heparin (6000 units/day) administration on days 1–28. The neoadjuvant chemoradiotherapy schedule for 2010–2017 comprised S-1-based chemotherapy and concurrent radiotherapy (40.0 Gy in 20 fractions). S-1 (60 mg/m^2^) was administered orally on the day of radiotherapy, while the other drugs were administered as previously reported. The duration of these regimens was 28 days.

In addition to these protocols, physicians selected regimens based on their preferences, including the use of 5-fluorouracil alone, S-1 alone, gemcitabine alone, and gemcitabine combined with S-1 or nab-paclitaxel.

After completing neoadjuvant chemoradiotherapy, patients were re-evaluated using contrast-enhanced multidetector CT. Patients without evidence of progressive disease or distant metastasis approximately 2 weeks after completing neoadjuvant chemoradiotherapy were considered candidates for curative-intent surgery.

Postoperative chemotherapy mainly comprised S-1 for 6 months.^14^

### Surgical Procedure

Pylorus-preserving pancreatoduodenectomy, subtotal stomach-preserving pancreatoduodenectomy, distal pancreatectomy, or total pancreatectomy with extensive lymphatic and connective tissue clearance were performed.^15, 16^

### Pathological Evaluation

Pathological stage was determined using the General Rules for the Study of Pancreatic Cancer, 7th edition.^17^ Tumor size; differentiation; main pancreatic duct, serosal side of the anterior pancreatic tissue, retropancreatic tissue, distal bile duct, duodenum, PV, SMV, common hepatic artery, superior mesenteric artery, celiac artery, and extrapancreatic nerve plexus invasion; lymph node metastasis; and surgical margins were evaluated. Surgical margins, evaluated using intraoperative frozen sections of the pancreas and bile ducts, were considered positive when cancer cells were present at the resection lines or in the dissected peripancreatic margin; the 0 mm rule was applied. Neoadjuvant therapy efficacy was evaluated by assessing residual viable tumor cells using the College of American Pathologists grading system.^18^

### Follow-Up

After discharge, patients underwent 3-monthly outpatient follow-up. Clinical and laboratory evaluations and multidetector CT were performed at each 3- to 6-monthly visit at the outpatient physician’s discretion.

A multidisciplinary team comprising hepatobiliary-pancreatic surgeons, internal medicine specialists, and a radiologist confirmed recurrence on suspicion. The recurrence pattern was assessed considering only the first site. Locoregional recurrence was defined as recurrence in the remnant pancreas or surgical bed, such as the soft tissue along the celiac or superior mesenteric artery, or around the pancreatojejunostomy site. Distant recurrence was stratified into liver, lung, and peritoneal metastases, defined as masses on CT during postoperative follow-up. Radiologically suspected recurrence did not require histological confirmation.

### Statistical Analyses

Continuous variables are presented as medians with interquartile ranges (IQRs), and categorical variables as frequencies and percentages. Clinicopathological findings were compared using the Mann–Whitney *U* test for continuous variables and the Chi-square or Fisher’s exact test for categorical variables. A minimal *p*-value approach was used to determine the optimal cut-offs for continuous variables, such as age, radiological tumor size, albumin level, neutrophil-to-lymphocyte ratio, CA19-9 level, and DUPAN-2 level.^19, 20^ Recurrence-free survival (RFS) was calculated from the date of surgery to the date of recurrence confirmation or last follow-up, and overall survival (OS) was calculated using the Kaplan–Meier method from the date of treatment initiation to the date of last follow-up in an intention-to-treat analysis; the survival curves were compared using the log-rank test. The prognostic factors for intention-to-treat OS in the upfront surgery group were investigated using multivariable analysis with a Cox proportional hazards model. Significant factors (*p* < 0.05) in the univariate analysis were entered into the multivariate model. Each significant independent prognostic factor was scored as 0 or 1, and the prognostic score was calculated by adding these scores.

The level of significance was set at *p* < 0.05. All analyses were performed using JMP^®^ Pro 17 (SAS Institute Inc., Cary, NC, USA).

## Results

### Participant Characteristics

**Fig. 1:**
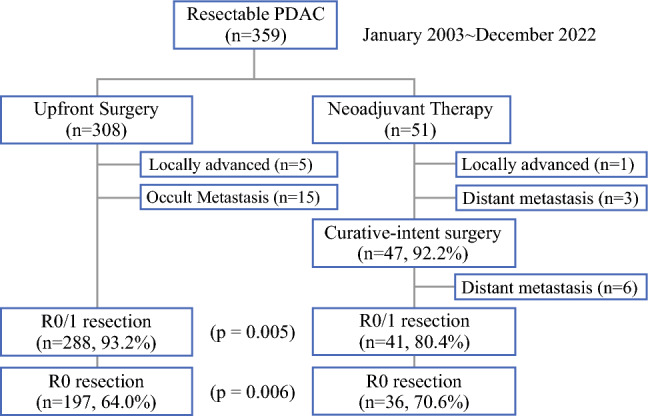
Study flowchart. *PDAC* pancreatic ductal adenocarcinoma

Figure 1 shows the study flowchart. Among the 359 included patients, 308 (85.8%) and 51 (14.2%) underwent upfront surgery and received neoadjuvant therapy, respectively.

Five patients in the upfront surgery group had laparotomy diagnosed locally advanced cancer, and 15 had radiologically detected occult metastasis. In the neoadjuvant therapy group, one patient had local progression, three had distant metastasis at follow-up, and six had laparotomy detected distant metastasis.

In the intention-to-treat analysis, the upfront surgery group (288/308, 93.2%) had a significantly higher R0/1 resection rate than the neoadjuvant therapy group (41/51, 80.4%; *p* = 0.005). Conversely, the neoadjuvant therapy group (36/51, 70.6%) had a significantly higher R0 resection rate than the upfront surgery group (197/308, 64.0%; *p* = 0.006).

### Clinicopathological Features

Table 1 shows the clinicopathological features of the participants. The neoadjuvant therapy group was significantly younger (median 67 years [IQR 61–74] vs. upfront surgery: 72 years [IQR 64–77]; *p* = 0.016), had a significantly larger radiological tumor size at diagnosis (27 mm [IQR 20–32] vs. upfront surgery: 20 mm [IQR 15–25]; *p* < 0.001), and had a significantly higher CA19-9 level at diagnosis (93 U/mL [IQR 42–413] vs. upfront surgery: 68 U/mL [IQR 19–217]; *p* = 0.034) than the upfront surgery group.

**Table 1:** Patient clinicopathological characteristics

Variables	Upfront surgery [*n* = 308]	Neoadjuvant therapy [*n* = 51]	*p*-Value
*Demographics*			
**Age, years [median (IQR)]**	**72 (64–77)**	**67 (61–74)**	**0.016**
Sex			0.720
Male	179 (58.1)	31 (60.8)	
Female	129 (41.9)	20 (39.2)	
*Radiological findings*			
Tumor location			0.115
Head	169 (54.9)	34 (66.7)	
Body and tail	139 (45.1)	17 (33.3)	
**Tumor size, mm [median (IQR)]**	**20 (15–25)**	**27 (20–32)**	**<** **0.001**
Nodal involvement			0.181
Absent	264 (85.7)	40 (78.4)	
Present	44 (14.3)	11 (21.6)	
*Serum marker*			
Albumin, g/dL [median (IQR)]	4.0 (3.7–4.3)	4.0 (3.6–4.2)	0.946
*Systemic inflammation marker*			
Neutrophil-to-lymphocyte ratio [median (IQR)]	2.42 (1.78–3.40)	2.22 (1.65–3.09)	0.189
*Tumor marker*			
**CA19-9, U/mL [median (IQR)]**	**68 (19–217)**	**93 (42–413)**	**0.034**
DUPAN-2, U/mL [median (IQR)]	107 (27–448)	83 (38–507)	0.928
*Year of starting treatment*			
2003–2017	195 (63.3)	37 (72.5)	0.194
2017–2022	113 (36.7)	14 (27.5)	
*Regimen of neoadjuvant therapy*			
5-fluorouracil alone	–	4 (7.8)	–
S-1 alone	–	5 (9.8)	–
Gemcitabine alone	–	1 (2.0)	–
Gemcitabine + S-1	–	6 (11.8)	–
Gemcitabine + nab-paclitaxel	–	3 (5.9)	–
5-fluorouracil based neoadjuvant chemoradiotherapy	–	17 (33.3)	–
S-1 based neoadjuvant chemoradiotherapy	–	15 (29.4)	–
Duration of neoadjuvant therapy, months (IQR)	–	29 (28–55)	–
*Surgical procedure*^a^			
**Underwent resection**	**288 (93.5)**	**41 (80.4)**	**0.005**
Pancreaticoduodenectomy	146 (50.7)	25 (61.0)	0.464
Distal pancreatectomy	125 (43.4)	14 (34.1)	
Total pancreatectomy	17 (5.9)	2 (4.9)	
*Pathological findings*^a^			
Diagnosis at pathology			0.758
Invasive ductal carcinoma	280 (97.2)	40 (97.6)	
Acinar cell carcinoma	2 (0.7)	0 (0.0)	
Intraductal papillary mucinous neoplasm	6 (2.1)	1 (2.4)	
**Tumor size, mm [median (IQR)]**	**28 (21–35)**	**20 (16–29)**	**<** **0.001**
**Lymphovascular invasion**			**<** **0.001**
**Absent**	**54 (18.8)**	**20 (48.8)**	
**Present**	**234 (81.2)**	**21 (51.2)**	
**Perineural invasion**			**0.001**
**Absent**	**113 (39.2)**	**27 (65.9)**	
**Present**	**175 (60.8)**	**14 (34.1)**	
**Surgical margin status**			**0.006**
**R0**	**197 (68.4)**	**36 (87.8)**	
**R1**	**91 (31.6)**	**5 (12.2)**	
T status			0.821
0	2 (0.7)	0 (0.0)	
1	20 (6.9)	4 (9.8)	
2	8 (2.8)	1 (2.4)	
3	258 (89.6)	36 (87.8)	
N status			0.155
0	81 (28.1)	16 (39.0)	
1a	121 (42.0)	18 (43.9)	
1b	86 (29.9)	7 (17.1)	
*Pathological response, CAP grades*^b^			
0	–	1 (2.4)	–
1	–	2 (4.9)	–
2	–	15 (36.6)	–
3	–	23 (56.1)	–
*Postoperative findings*^a^			
Administration of adjuvant chemotherapy	212 (73.6)	29 (70.7)	0.699

Data are expressed as *n* (%) unless otherwise specified

*IQR* interquartile range, *CA19-9* carbohydrate antigen 19-9, *DUPAN-2* Duke pancreatic monoclonal antigen type 2, *CAP* College of American Pathologists

Bold font indicates *p* < 0.05

^a^Patients who underwent pancreatectomy (*n* = 329)

^b^Patients who underwent neoadjuvant therapy and pancreatectomy, diagnosed with invasive pancreatic ductal carcinoma (*n* = 41)

Among patients who underwent R0/1 resection, the neoadjuvant therapy group had a smaller pathological tumor size (20 mm [IQR 16–29] vs. upfront surgery: 28 mm [IQR 21–35]; *p* < 0.001), lower lymphovascular invasion incidence (51.2% vs. upfront surgery: 81.2%; *p* < 0.001), lower perineural invasion occurrence (34.1% vs. upfront surgery: 60.8%; *p* = 0.001), and a lower positive surgical margin rate (12.2% vs. upfront surgery: 31.6%; *p* = 0.006) than the upfront surgery group.

### Intention-to-Treat Overall Survival (OS)

**Fig. 2:**
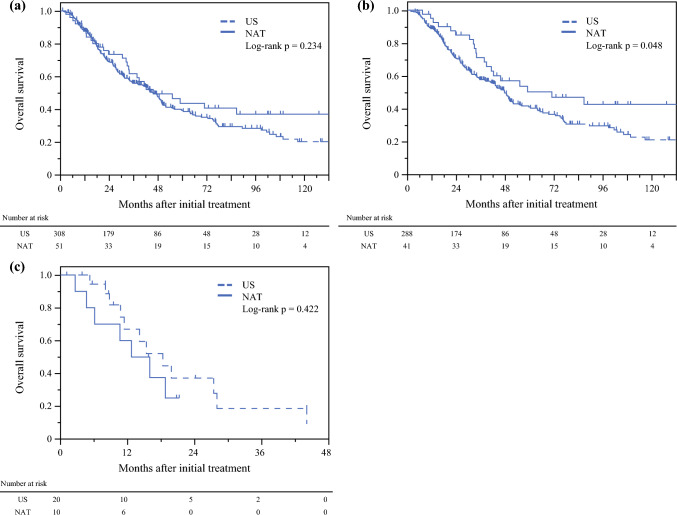
Kaplan–Meier curves of overall survival in the three groups of patients with resectable pancreatic ductal adenocarcinoma. **a** Patients in the intention-to-treat analysis (*n* = 359); **b** patients who underwent curative resection (*n* = 329); and **c** patients who did not undergo resection (*n* = 30). These groups have been stratified based on whether the patients underwent upfront surgery or received neoadjuvant therapy. *US* upfront surgery, *NAT* neoadjuvant therapy

The median intention-to-treat OS did not differ between the neoadjuvant therapy and upfront surgery groups (45.7 vs. 45.6 months; *p* = 0.234) (Fig. 2a). Among patients who underwent R0/1 resection, the neoadjuvant therapy group had better intention-to-treat OS than the upfront surgery group (71.0 vs. 47.7 months; *p* = 0.048) (Fig. 2b), with no significant difference between the neoadjuvant therapy and upfront surgery groups among patients who underwent non-curative resection (14.3 vs. 18.3 months; *p* = 0.422) (Fig. 2c).

### Prognostic Factors for OS in the Upfront Surgery Group

Table 2 show the results of the univariate and multivariate analyses, respectively. The optimal cut-offs of continuous variables were determined using the minimal *p*-value approach (see electronic supplementary material [ESM] Fig. 1, which shows the cut-offs). In the univariate analysis, age ≥ 75 years, tumor size ≥ 35 mm, albumin level ≤ 3.5 g/dL, neutrophil-to-lymphocyte ratio ≥ 3.5, CA19-9 level ≥ 250 U/mL, and DUPAN-2 level ≥ 750 U/mL were significant prognostic factors. In the multivariate analysis, age ≥ 75 years (hazard ratio [HR] 1.532, 95% confidence interval [CI] 1.110–2.133; *p* = 0.012), tumor size ≥ 35 mm (HR 1.974, 95% CI 1.238–3.146; *p* = 0.004), albumin level ≤ 3.5 g/dL (HR 2.042, 95% CI 1.134–3.103; *p* < 0.001), neutrophil-to-lymphocyte ratio ≥ 3.5 (HR 1.648, 95% CI 1.144–2.372; *p* = 0.007), CA19-9 level ≥ 250 U/mL (HR 1.628, 95% CI 1.136–2.333; *p* = 0.008), and DUPAN-2 level ≥ 750 U/mL (HR 1.738, 95% CI 1.178–2.565; *p* = 0.005) were independent prognostic factors.

**Table 2:** Univariate and multivariate Cox proportional regression model to predict intention-to-treat overall survival in patients with R-PDAC using factors at initial diagnosis

Variables	Univariate		Multivariable	
	HR (95% CI)	*p*-Value	HR (95% CI)	*p*-Value
*Demographics*				
**Age**				
**< 75 years**	**Ref**		**Ref**	
**≥ 75 years**	**1.647 (1.197–2.267)**	**0.002**	**1.532 (1.100–2.133)**	**0.012**
Sex				
Female	Ref			
Male	0.854 (0.629–1.160)	0.313		
*Radiological findings*				
Tumor location				
Head	Ref			
Body and tail	1.233 (0.909–1.673)	0.178		
**Tumor size**				
**< 35 mm**	**Ref**		**Ref**	
**> 35 mm**	**2.416 (1.540–3.791)**	**<** **0.001**	**1.974 (1.238–3.146)**	**0.004**
Nodal involvement				
Absent	Ref			
Present	1.112 (0.735–1.680)	0.616		
*Serum marker*				
**Albumin**				
**> 3.5 g/dL**	**Ref**		**Ref**	
**≤ 3.5 g/dL**	**2.390 (1.599–3.571)**	**< 0.001**	**2.042 (1.344–3.103)**	**< 0.001**
*Systemic inflammation marker*				
**Neutrophil-to-lymphocyte ratio**				
**< 3.5**	Ref		Ref	
**≥ 3.5**	**1.587 (1.108–2.273)**	**0.012**	**1.648 (1.144–2.372)**	**0.007**
*Tumor marker*				
**CA19-9**				
**< 250 U/mL**	**Ref**		**Ref**	
**≥ 250 U/mL**	**2.044 (1.450–2.879)**	**<** **0.001**	**1.628 (1.136–2.333)**	**0.008**
**DUPAN-2**				
**< 750 U/mL**	**Ref**		**Ref**	
**≥ 750 U/mL**	**1.821 (1.260–2.631)**	**0.001**	**1.738 (1.178–2.565)**	**0.005**
*Year of starting treatment*				
2003–2017	Ref			
2017–2022	0.963 (0.672–1.380)	0.837		

*HR* hazard ratio, *CI* confidence interval, *CA19-9* carbohydrate antigen 19-9, *DUPAN-2* Duke pancreatic monoclonal antigen type 2, *Ref* reference

Bold font indicates *p* < 0.05

### Prognostic Score-Based Stratification in the Upfront Surgery Group

**Fig. 3:**
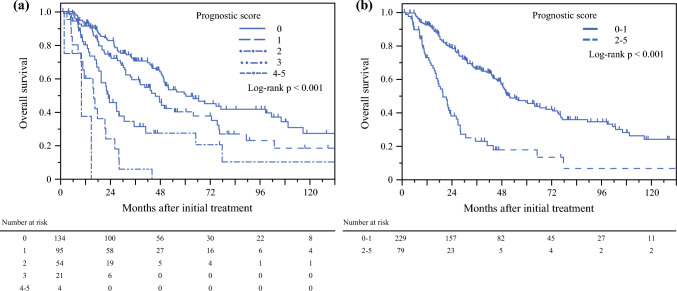
Kaplan–Meier curves of overall survival in patients with resectable pancreatic ductal adenocarcinoma who underwent upfront surgery, with stratification based on the prognostic score. **a** Prognostic score < 2; **b** prognostic score ≥ 2. *US* upfront surgery

With the exception of those aged ≥ 75 years, patients were categorized based on the number of poor prognostic factors, as age was not considered when determining the suitability of neoadjuvant therapy. Figure 3a shows the Kaplan–Meier curves of intention-to-treat OS. The median intention-to-treat OS in the upfront surgery group was 60.4, 45.6, 22.9, 15.9, and 10.0 months for patients with prognostic scores of 0, 1, 2, 3, and 4–5, respectively (HRs 1.00, 1.49, 2.83, 7.40, and 19.0, respectively). Patients with a prognostic score < 2 had significantly better prognosis than those with a prognostic score ≥ 2 (59.0 vs. 22.9 months; *p* < 0.001) (Fig. 3b).

Compared with patients who underwent curative resection with a prognostic score < 2, those with a prognostic score ≥ 2 had a significantly shorter RFS (26.6 vs. 8.9 months; *p* < 0.001) [see ESM Fig. 2a, which shows the RFS], significantly higher cumulative locoregional recurrence incidence (not reached vs. 24.2 months; *p* < 0.001) [see ESM Fig. 2b, which shows the cumulative locoregional recurrence incidence], and significantly higher cumulative distant metastasis incidence (58.3 vs. 13.0 months; *p* < 0.001) [see ESM Fig. 2c, which shows the cumulative distant metastasis incidence].

### Intention-to-Treat OS According to Prognostic Score

**Fig. 4:**
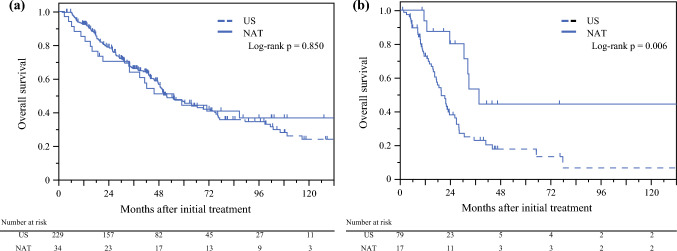
Kaplan–Meier curves of overall survival in groups according to the prognostic score. **a** Prognostic score < 2; **b** prognostic score ≥ 2. The groups are those in the intention-to-treat analysis, with stratification based on whether patients underwent upfront surgery or received neoadjuvant therapy. *US* upfront surgery, *NAT* neoadjuvant therapy, *PS* prognostic score

Among patients with prognostic scores of 0 and 1, the intention-to-treat OS did not significantly differ between the neoadjuvant therapy and upfront surgery groups (55.1 vs. 51.7 months; *p* = 0.850) (Fig. 4a). Among patients with a prognostic score ≥ 2, the neoadjuvant therapy group had better intention-to-treat OS than the upfront surgery group (37.7 vs. 19.7 months; *p* = 0.006) (Fig. 4b).

## Discussion

We compared the long-term outcomes of upfront surgery and neoadjuvant therapy in patients with resectable PDAC. Neoadjuvant therapy was associated with a higher R0 resection rate, smaller pathological tumor size, and lower lymphovascular and perineural invasion rates than upfront surgery; however, intention-to-treat OS did not differ between these groups. Therefore, neoadjuvant therapy may not benefit all patients with resectable PDAC. We also identified independent prognostic factors in the upfront surgery group, including tumor size ≥ 35 mm, CA19-9 level ≥ 250 U/mL, DUPAN-2 level ≥ 750 U/mL, albumin level ≤ 3.5 g/dL, and neutrophil-to-lymphocyte ratio ≥ 3.5. These factors can be easily measured and clinically addressed. In terms of resectable PDAC categorization, the tumor size represented the anatomical classification, CA19-9 and DUPAN-2 levels represented the biological classification, and the albumin level and neutrophil-to-lymphocyte ratio represented the conditional classification. Furthermore, we demonstrated that a clinically significant prognostic scoring system can aid long-term outcome stratification in patients with resectable PDAC. Specifically, in high-risk patients (prognostic score ≥ 2), neoadjuvant therapy may improve long-term survival. We believe this is the first study to identify biological and conditional factors of resectable PDAC. The prognostic scoring system may be valuable for selecting patients who will benefit from neoadjuvant therapy.

Neoadjuvant therapy yielded benefits in patient selection and local control. The neoadjuvant therapy group had significantly lower curative-intent surgery (92.2%) and higher R0/1 resection (80.4%) rates than the upfront surgery group. In previous randomized clinical trials, curative-intent surgery and R0/1 resection were performed in approximately 69–75% and 61–72% of patients with resectable PDAC, respectively.^4, 5, 21^ The between-group difference in the R0/1 resection rate can be attributed to patients experiencing progressive disease during neoadjuvant therapy. Among patients who did not undergo R0/1 resection, the intention-to-treat OS did not differ between the neoadjuvant therapy and upfront surgery groups, with the neoadjuvant therapy group showing a median intention-to-treat OS of only 14.3 months. Consequently, we hypothesized that these patients showed a lack of benefit from neoadjuvant therapy because of an aggressive tumor biology rather than a missed opportunity for curative-intent surgery.

In terms of local control, the neoadjuvant therapy group had a significantly higher R0 resection rate than the upfront surgery group, indicating that neoadjuvant therapy might aid the identification of patients who would truly benefit from curative-intent surgery and are candidates for R0 resection. Furthermore, pathological evaluation revealed that the neoadjuvant therapy group exhibited a tumor size reduction and decrease in microscopic invasion into the surrounding tissue, including lymphovascular and perineural invasion. Achieving R0 resection is a significant prognostic factor in patients with PDAC.^22^ Local control through neoadjuvant therapy may contribute to improved prognosis.

In this study, we identified five biological and conditional markers evaluated at diagnosis. The prognostic scoring system effectively reflected survival in patients with resectable PDAC. Interestingly, patients with resectable tumors in the upfront surgery group and prognostic scores of 3 and 4–5 showed median intention-to-treat OS values of 15.9 and 10.0 months, respectively, which were worse than those of patients with unresectable advanced PDAC in a previous report.^23^ Additionally, upfront surgery is unsuitable for high-risk patients with resectable PDAC owing to the invasiveness of laparotomy and delay in initiating systemic therapy. Consequently, the anatomical classification alone may be inadequate for determining tumor aggressiveness and determining the optimal treatment strategy. Incorporating biological and conditional factors can improve decision making. In this study, patients with a prognostic score ≥ 2 who underwent curative resection had a higher likelihood of experiencing locoregional recurrence and distant metastasis than those with a prognostic score < 2 (see ESM Fig. 2b, which shows the cumulative locoregional recurrence incidence). Within the same resectability category, the local tumor aggressiveness varied, potentially leading to R1 resection and locoregional recurrence. Furthermore, the high incidence of early distant metastasis in patients with a prognostic score ≥ 2 suggested the possible presence of micrometastases at the time of surgery.

In this study, the intention-to-treat OS did not differ significantly between the neoadjuvant therapy and upfront surgery groups. In the Prep-02/JSAP-05 study, neoadjuvant therapy with two cycles of gemcitabine plus S-1 showed a survival benefit; however, resectability was only defined based on arterial invasion. Meanwhile, the PREOPANC^4^ and SWOG S1505^5^ trials did not demonstrate a survival benefit of neoadjuvant therapy in patients with resectable PDAC. Specifically, in patients with a prognostic score of 0–1, the intention-to-treat OS did not differ between the neoadjuvant therapy and upfront surgery groups. However, in patients with a prognostic score ≥ 2, those who underwent neoadjuvant therapy had a significantly longer intention-to-treat OS than those who underwent upfront surgery, suggesting that neoadjuvant therapy may offer a survival benefit in high-risk patients with resectable PDAC, whereas upfront surgery may be a suitable option for low-risk patients. Physicians typically perform pathological evaluations before starting neoadjuvant therapy; however, needle-tract peritoneal dissemination resulting from endoscopic ultrasound aspiration has been reported.^24^ Considering its drawbacks, neoadjuvant therapy is not recommended for all patients with resectable PDAC.

The preoperative CA19-9 level is a known prognostic factor in patients with PDAC; as the CA19-9 level increases, the resection and survival rates decrease.^25^ In patients with anatomically resectable PDAC, the reported CA19-9 cut-off level ranges from 120 to 500 U/mL.^26-28^ In this study, we proposed an optimal CA19-9 cut-off level of 250 U/mL. Nevertheless, the CA19-9 level alone cannot predict the prognosis of all patients with PDAC, as 10% of these patients showed no CA19-9 secretion. The serum tumor marker DUPAN-2 has attracted attention as a precursor of CA19-9 in patients with PDAC. DUPAN-2 might reflect tumor aggressiveness in patients with PDAC who do not secrete CA19-9^29^ because it is not converted to CA19-9 in patients who lack fucosyltransferase.^29, 30^ A previous study demonstrated a positive correlation between CA19-9 and DUPAN-2 levels in the elevated CA19-9 group, and a weak negative correlation between these factors in the normal CA19-9 group.^31^ This may be influenced by the amount of Lewis antigen produced by the tumor and Lewis enzyme activity. We found DUPAN-2 and CA19-9 levels to be independent prognostic factors. Our suggested optimal DUPAN-2 cut-off level is 750 U/mL, whereas cut-off values ranging from 150 to 2000 U/mL have been previously reported.^29, 31, 32^ The wide range of cut-off values in previous studies reflects the heterogeneity in the study populations. We believe this is the first study to evaluate the prognostic impact of the DUPAN-2 level at diagnosis in a cohort comprising only patients with resectable PDAC.

Currently, pathological staging is performed according to the tumor size classification; however, the evidence for the cut-off used is unclear.^17^ Using the minimal *p*-value approach, we found that a radiological tumor size cut-off value of 35 mm was optimal for patients with resectable PDAC. This value was comparable with that in previous research, and a preoperative tumor size ≥ 35 mm was a predictor of R1 resection.^33^ Additionally, albumin level^34^ and neutrophil-to-lymphocyte ratio^35^ are well-known prognostic factors for PDAC.

This study has some limitations. First, this was a retrospective, single-center study, which may limit the generalizability of the findings. Additionally, due to the small size of the neoadjuvant therapy group, the subgroup analysis was not robust and drawing definitive conclusions was challenging. Second, the sample size was small; therefore, our findings might lack validity. A multicenter study should be conducted to validate the findings. Third, selection bias might have occurred as patients were not randomly allocated to the groups, and allocation was based on the physician’s judgment and the patient’s preference. Fourth, this study was conducted over 20 years, during which the treatment strategies, such as surgical techniques, perioperative management, and chemotherapy drugs, varied. However, treatment duration was not a significant independent poor prognostic factor in our multivariate analysis. Although neoadjuvant chemotherapy consisting of gemcitabine plus S-1 has been recommended for resectable PDAC in Japan,^17^ the optimal regimen remains uncertain. Fifth, the final pathological evaluation revealed that approximately 3% of patients had a pathology other than pancreatic invasive ductal carcinoma; this reflects diagnostic difficulties, although the proportion herein was less than that in previous randomized clinical trials.^4^

## Conclusion

Neoadjuvant therapy could aid patient selection and local control in patients with resectable PDAC; however, its clinical benefits in terms of long-term outcomes may not be applicable to all patients with resectable PDAC. Nevertheless, neoadjuvant therapy can improve long-term outcomes in select patients with resectable PDAC who possess high-risk biological and conditional factors (prognostic score ≥ 2).

## Supplementary Information

Below is the link to the electronic supplementary material.

Supplementary Fig. 1 Optimal cut-off values with the respective *p*-values for (**a**) age, (**b**) tumor size, (**c**) carbohydrate antigen 19-9 level, (**d**) Duke pancreatic monoclonal antigen type 2 level, (**e**) albumin level, and (**f**) neutrophil-to-lymphocyte ratio. *CA19-9* carbohydrate antigen 19-9, *DUPAN-2* Duke pancreatic monoclonal antigen type 2, *NLR* neutrophil-to-lymphocyte ratio (TIFF 47848 kb)

Supplementary Fig. 2 Kaplan–Meier curves of (**a**) recurrence-free survival, (**b**) cumulative incidence of locoregional recurrence, and (**c**) distant metastasis, stratified based on the prognostic score (< 2 or ≥ 2) among patients who underwent curative resection in the upfront surgery group (TIFF 47848 kb)
