# Supplementary figures and images for: Clinical Impact of Neoadjuvant Therapy for Resectable Pancreatic Ductal Adenocarcinoma: A Single-Center Retrospective Study
Source: Ann Surg Oncol. 2025 Jan 23;32(4):2830–40. doi: 10.1245/s10434-024-16851-z (PMC11882687; doi:10.1245/s10434-024-16851-z)

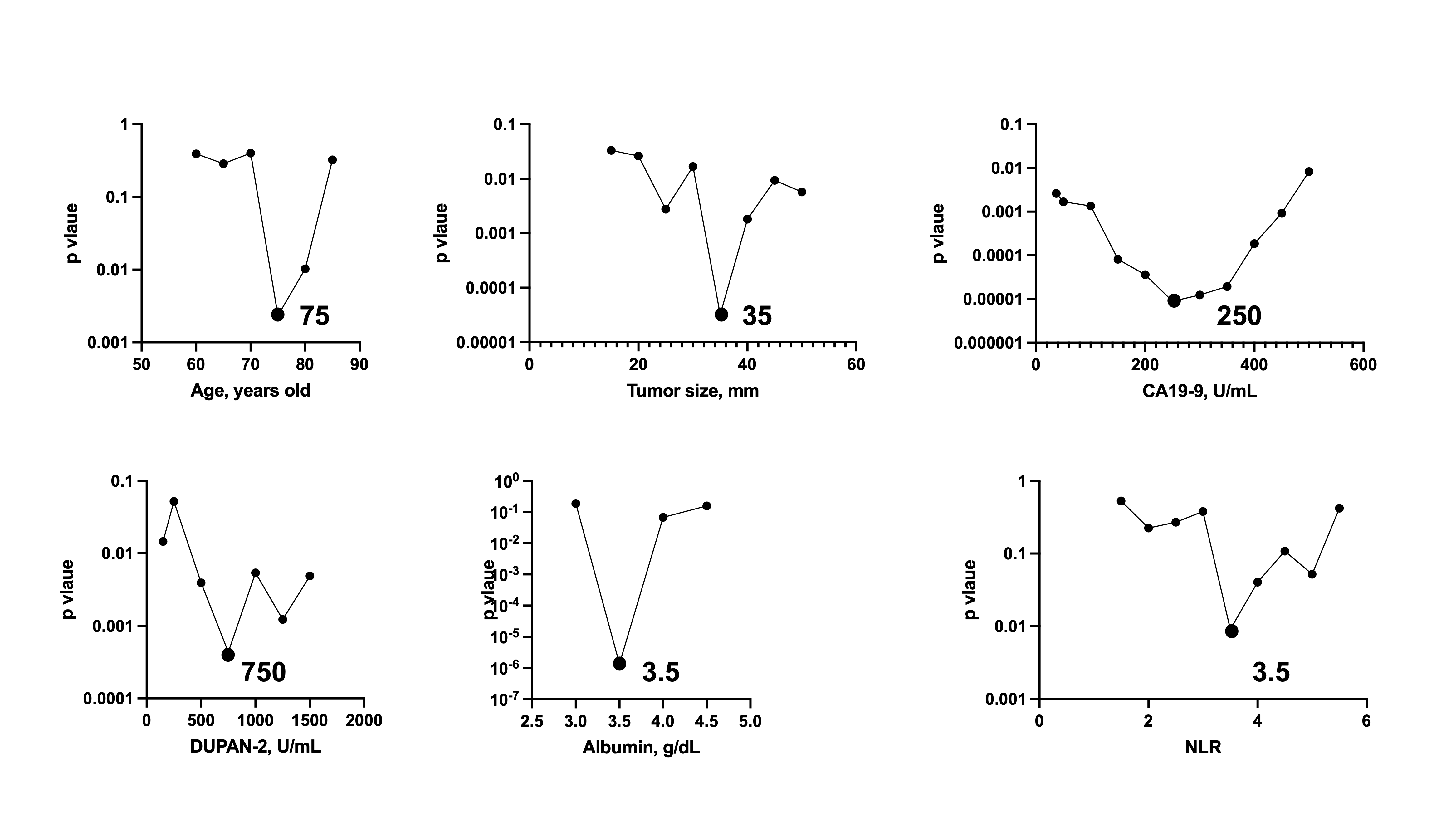

Supplement: Supplementary file 1 — Supplementary Fig. 1 Optimal cut-off values with the respective p-values for (a) age, (b) tumor size, (c) carbohydrate antigen 19-9 level, (d) Duke pancreatic monoclonal antigen type 2 level, (e) albumin level, and (f) neutrophil-to-lymphocyte ratio. CA19-9 carbohydrate antigen 19-9, DUPAN-2 Duke pancreatic monoclonal antigen type 2, NLR neutrophil-to-lymphocyte ratio (TIFF 47848 kb) [file 10434_2024_16851_MOESM1_ESM.tiff]

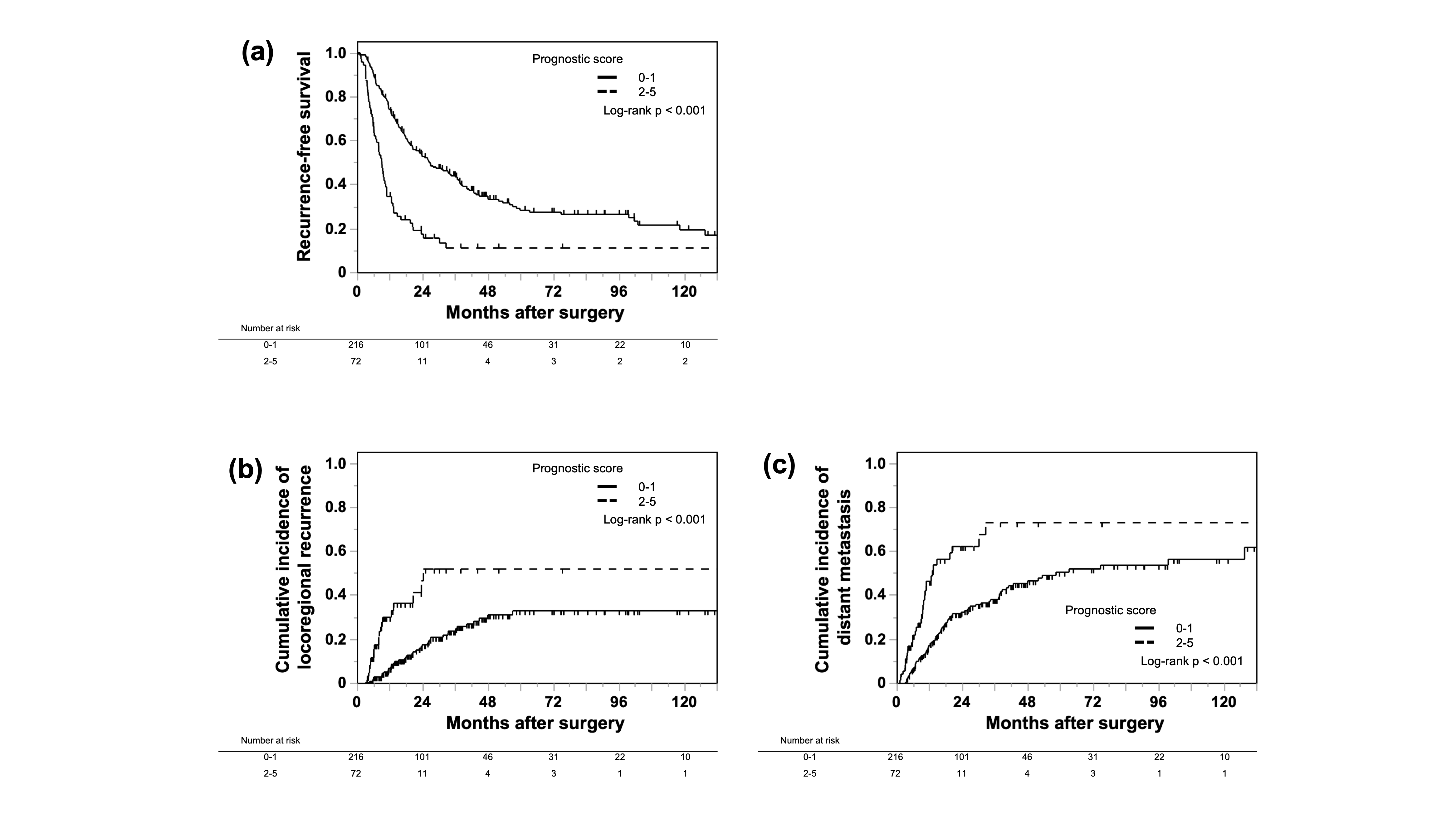

Supplement: Supplementary file 2 — Supplementary Fig. 2 Kaplan–Meier curves of (a) recurrence-free survival, (b) cumulative incidence of locoregional recurrence, and (c) distant metastasis, stratified based on the prognostic score (< 2 or ≥ 2) among patients who underwent curative resection in the upfront surgery group (TIFF 47848 kb) [file 10434_2024_16851_MOESM2_ESM.tiff]
